# Interobserver Variation Within Planning Target Volume and Organs at Risk in a Patient with Oropharyngeal Carcinoma: A Contouring Study with Anatomical Analysis

**DOI:** 10.3390/curroncol33010039

**Published:** 2026-01-11

**Authors:** Fabian Baier, Oliver Koelbl, Felix Steger, Isabella Gruber, Christoph Suess

**Affiliations:** Klinik und Poliklinik für Strahlentherapie, Universitätsklinikum Regensburg, 93053 Regensburg, Germanyc.suess@ukr.de (C.S.)

**Keywords:** oropharyngeal carcinoma, head and neck cancer, planning target volume, interobserver variability, organs at risk, contouring

## Abstract

**Background:** Despite the availability of contouring guidelines and advanced imaging modalities, interobserver variability (IOV) in the delineation of the planning target volume and organs at risk remains a critical factor influencing treatment quality in radiotherapy. The aim of this study was to examine variations in contour delineation with respect to anatomical landmarks, as well as differences in the inclusion of lymph node levels within the PTV. **Methods:** Ten senior radiation oncologists from six different institutions participated in the study and contoured PTV1, PTV2 and 16 OARs in a patient with oropharyngeal carcinoma. Interobserver variation was quantified by volume statistics such as mean, standard deviation (SD) and ranges, as well as using coefficient of variance (CoV) and conformity index (CI). **Results:** High agreement was observed in the inclusion of the ipsilateral lymph node levels Ib–IVa and VIIa+b, whereas notable discrepancies were identified in the delineation inclusion of the cervical triangle group and lateral supraclavicular nodes. Regarding OARs, the greatest variability was observed in the delineation of the left and right inner ear, with volume ranges of 0.12–2.84 cm^3^ and 0.11–2.38 cm^3^, respectively. **Conclusions:** This study reaffirms the presence of significant interobserver variability in the delineation of PTVs and OARs in patients with oropharyngeal carcinoma. Especially inclusion of elective lymph node levels and definition of margins around the gross tumor volume are substantial factors for IOV. By emphasizing structured anatomical assessment as a standard approach, variability can be minimized, treatment consistency enhanced, and ultimately, patient outcomes improved.

## Simple Summary

This study examined differences in the contouring of planning target volumes (PTVs) and organs at risk (OARs)—a critical component of modern radiotherapy planning—among senior radiation oncologists. Ten participants independently contoured a case of oropharyngeal carcinoma, revealing substantial discrepancies, including up to nearly two-fold differences in PTV size. These findings underscore the ongoing need to refine and standardize contouring practices to ensure greater consistency in radiotherapy planning and ultimately improve the quality and safety of patient care.

## 1. Introduction

Accurate delineation of planning target volumes (PTVs) and organs at risk (OARs) is a critical component of modern radiotherapy planning, impacting treatment efficacy and toxicity. However, interobserver variability (IOV) in contouring remains a significant challenge [1], contributing to inconsistencies in dose delivery and potentially affecting clinical outcomes. Previous studies have demonstrated substantial IOV in both target volume and OAR delineation [2,3,4], with deviations from established contouring protocols in head and neck cancer treatment being linked to poorer survival outcomes and increased risk of recurrence [5]. Additionally, the impact of different imaging modalities such as Computed Tomography (CT), Positron Emission Tomography-Compute Tomography (PET-CT) or Magnetic Resonance Imaging (MRI) on IOV has been subject of research [6].

Despite efforts to standardize delineation through consensus guidelines and atlases, variation persists due to multiple factors, including differences in clinical experience, interpretation of imaging modalities, and individual preferences regarding safety and set-up margins. While multiple studies have analyzed IOV using quantitative metrics such as the Dice similarity coefficient (DSC) and mean absolute surface distance (MASD) [7], these methods primarily focus on volumetric discrepancies rather than the anatomical reasoning behind them.

The aim of this study is to assess IOV in the delineation of target volumes and OARs, with an emphasis on anatomical factors influencing contouring differences. By evaluating discrepancies in the context of anatomical structures, this study seeks to provide insights that could contribute to improved standardization and adherence to guidelines. For target volume delineation, the DAHANCA recommendations were used as a reference [8], while considering OAR contouring consensus guidelines by Nielsen et al. [9] and Brouwer et al. [10] were implemented.

## 2. Materials and Methods

### 2.1. Acquisition of Data

The selected case in this study involved a patient with oropharyngeal p16-negative squamous cell carcinoma affecting the right soft palate and tonsil, accompanied by two ipsilateral lymph node metastases (clinical staging: cT3 cN2b cM0, UICC IVa). Patient file included reports from panendoscopy, a planning CT scan, a contrast-enhanced CT scan, a PET-CT scan, histopathological report and the tumor board decision. The diagnostic CT report described the primary tumor as difficult to circumscribe but identified two ipsilateral lymph node metastases in Level IIa and IIb, measuring 1.5 cm and 1.3 cm in diameter, respectively. No additional suspicious lymph nodes were detected in either the CT or PET scan. Panendoscopy revealed that the primary tumor extended from the right soft palate and tonsil to the posterior floor of the mouth, partially infiltrating the right base of the tongue. No suspicious lesions were found in the nasopharynx, posterior pharyngeal wall, or supraglottic region. Given the tumor’s extension to the base of the tongue and soft palate, it was classified as a midline tumor.

The planning CT scan was acquired with a slice thickness of 4 mm, with the patient positioned supine and without the administration of contrast agents (Scan range: 332.0 mm, kVp: 120, Canon Medical Systems, Neuss, Germany, CT Scanner Aquilion, Model TSX-201A).

Ten senior radiation oncologists (ROs) from six different institutions within the Qua-litätszirkel Strahlentherapie Ostbayern, each with a minimum of ten years of clinical experience, participated in this study. All participants received the same anonymized CT dataset, which did not include any pre-contoured structures. To support anatomical delineation, the contrast-enhanced diagnostic CT scan and the PET-CT scan was provided as supplementary imaging modality.

Each participant was instructed to manually delineate on the planning CT one target volume for primary tumor and low-risk elective nodal region (PTV1), a second target volume for the primary tumor and high-risk volume (PTV2) as well as the following OARs: brainstem, pituitary gland, inner ears, optic chiasm, spinal canal, optic nerves, eye lenses, temporomandibular joints (TMJ), parotid glands, and submandibular glands. No auto-contouring tools were used by the participants, they were not asked to use a specific contouring guideline, neither for PTVs nor OARs. In this study, the evaluation was intentionally performed on PTVs. While contouring guidelines and most studies on IOV primarily refer to CTV definitions, analyzing PTVs allowed us to capture both the anatomical and practical aspects of interobserver variation, including margin application and treatment geometry.

For analysis, the delineated contours from the various treatment planning systems were imported into the Monaco treatment planning system (Version 5.51.10, Elekta AB, Stockholm, Sweden) for standardized evaluation.

### 2.2. Measuring Interobserver Variation

To assess the level of agreement for each contour, the mean volume, standard deviation (SD), and coefficient of variation (CoV) were calculated, with the CoV defined as the ratio of the SD to the mean. Given the DSC’s sensitivity to the number of participants, the conformity index pairs (CIpairs) as described by Kouwenhoven et al. [11], was employed to evaluate PTV conformity.

For the anatomical analysis of contouring variability, specific reference points were measured, including the cranial and caudal borders of the PTV1, as well as its width on a representative axial slice. The floor of the sphenoid sinus was defined as the cranial reference point of the PTV1, while the sternoclavicular joints served as the caudal landmark.

A key focus of the analysis was the inclusion of lymph node levels within the PTV. Lymph node levels were contoured according to the guidelines described by Grégoire et al. [12]. Subsequently, the intersection between the PTV and each lymph node level was calculated. A lymph node level was considered included in the PTV if the overlapping volume accounted for more than 70% of its total volume, following the criteria established by Vorwerk et al. [13].

## 3. Results

Among the ten radiation oncologists (ROs) that participated in the study, two were affiliated with a university hospital, four worked in public hospitals, and four practiced in private institutions. Overall, six participants delineated all requested structures, while the remaining four omitted certain volumes.

All participants delineated both the primary tumor and low-risk elective nodal region (PTV1) as well as the boost volume compromised by Primary tumor and high-risk volume (PTV2). As shown in Table 1, the mean PTV1 volume was 855 cm^3^ (range: 687–1098 cm^3^) with a CoV of 0.16 and a pairwise conformity index (CIpairs) of 0.63. The mean PTV2 was 386 cm^3^ (range: 87–659 cm^3^), exhibiting a higher CoV of 0.55 and a lower CIpairs of 0.42, indicating greater variability in boost volume delineation.

**Figure 1:**
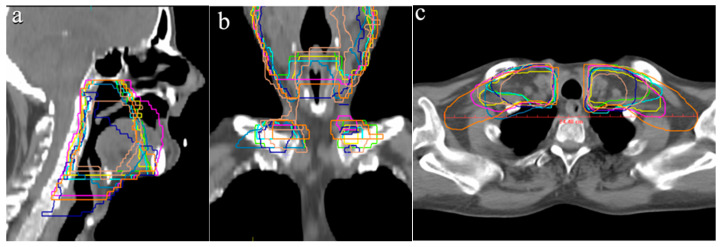
Variation of PTV1 outlines: (**a**) cranial border in relation to the floor of the sphenoid sinus, (**b**) caudal border and (**c**) width of PTV1. Different colored lines represent the PTV outlines of the respective participants.

Regarding the cranial border of the PTV1, variability of up to 8 mm was observed, with seven participants selecting the same CT slice, positioned 4 mm below the floor of the sphenoid sinus (Figure 1a). The caudal PTV1 border showed greater variation, with differences of up to 20 mm, while the average lower boundary was 12 mm above the sternoclavicular joint (Figure 1b). In a representative supraclavicular lymphatic drainage slice, the PTV1 width ranged from 7.0 cm to 24.4 cm, with a mean of 16.2 cm (Figure 1c). Due to the location of tumor, the tongue was included in the PTV1 by all participants, with more than two-thirds of its volume consistently delineated.

With respect to lymphatic drainage, all ROs included both ipsilateral and contralateral sites. When analyzing lymph node levels individually, high concordance was observed for the ipsilateral submandibular, upper and lower jugular, retropharyngeal, and retrostyloid lymph node levels, which were contoured by all participants (Table 2). However, considerable discrepancies were noted for the submental lymph nodes and ipsilateral levels IVb, Va, Vb, and Vc, which were delineated by only three to five participants.

For OARs, the highest CoV was recorded for the pituitary gland, followed by the inner ear and optic chiasm, indicating significant variability in their delineation. In contrast, the submandibular glands and brainstem were contoured with the highest consistency among participants (Table 3).

**Figure 2:**
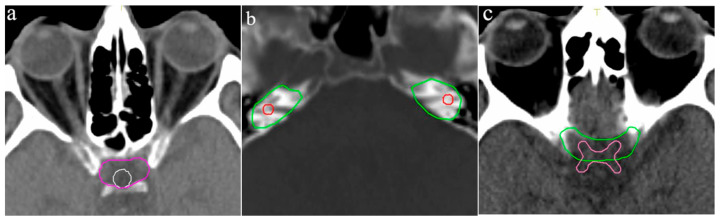
Variation in OAR outlines: (**a**) Pituitary gland, (**b**) Left and right inner ear, (**c**) optic chiasm. Different colored lines represent the OAR outlines of the respective participants.

The pituitary gland was delineated by only seven ROs and exhibited the highest CoV (0.82, Range: 0.18–1.96 cm^3^). The largest volume resulted from cranial extension beyond the sella turcica, incorporating portions of the optic chiasm and the middle cerebral artery (Figure 2a). Conversely, the smallest volume was delineated on a single CT slice.

The left and right inner ear had a CoV of 0.72 and 0.68, respectively, with volume ranges of 0.12–2.84 cm^3^ and 0.11–2.38 cm^3^, respectively. One participant included the entire petrous part of the temporal bone, leading to the largest delineated volume, while another contoured only a small central portion of the inner ear on two CT slices (Figure 2b).

The optic chiasm was delineated by nine of the ten participants, with a mean volume of 1.80 cm^3^ (Range: 0.36–3.62 cm^3^, CoV: 0.67). Variability was primarily caused by the addition of margins to the OAR, leading to the inclusion of portions of the optic nerves and adjacent vascular structures, such as the anterior and middle cerebral arteries (Figure 2c). Four participants applied such margin to the optic chiasm measuring up to 6 mm.

Left and right eye lenses were delineated by all participants with a moderate variability (Range: 0.14–0.52 cm^3^ and 0.15–0.52 cm^3^, CoV: 0.50 and 0.53, respectively).

**Figure 3:**
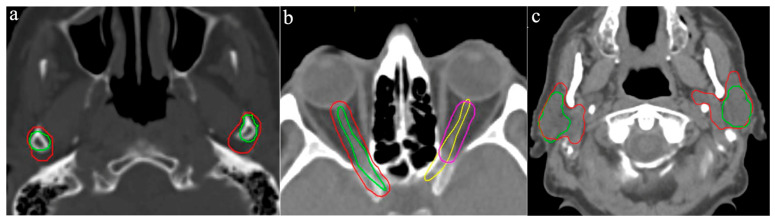
Variation in OAR outlines: (**a**) temporomandibular joints, (**b**) optical nerves, (**c**) left and right parotid glands. Different colored lines represent the OAR outlines of the respective participants.

Left and right TMJs were delineated by eight participants, showing moderate variability (Range: 1.22–3.03 cm^3^ and 1.02–3.16 cm^3^, CoV: 0.32 and 0.39, respectively). Differences arose mainly from variations in the axial margins around the mandible (Figure 3a). Also, some participants did not include the superior portion of the joint within the temporal bone.

The left and right optic nerves were contoured by all participants, yielding mean volumes of 1.60 cm^3^ and 1.70 cm^3^ respectively (Range: 1.00–2.26 cm^3^ and 1.09–2.96 cm^3^, CoV: 0.34 and 0.32, respectively). While all ROs began contouring at the posterior edge of the eyeball, some did not extend the structure to fully connect with the optic chiasm. The largest volumes resulted from the addition of extensive margins up to 4 mm, producing contours up to 10 mm wide (Figure 3b).

The left and right parotid glands were delineated by all participants, showing relatively high variability in volume (Range: 12.0–29.2 cm^3^ and 12.8–26.5 cm^3^, CoV: 0.30 and 0.24, respectively). Discrepancies were most pronounced in the ventral and medial extensions of the contours (Figure 3c).

The spinal canal was contoured by all participants, with a mean volume of 33.9 cm^3^ (Range: 22.7–45.2 cm^3^, CoV: 0.20). Cranio-caudal length varied substantially, ranging from 14.4 cm to 24.0 cm. The smallest volume resulted from delineation of only the spinal cord rather than the entire spinal canal.

Left and right submandibular glands were delineated by all participants showing modest IOV (Range: 5.54–10.4 cm^3^ and 6.67–11.8 cm^3^, CoV: 0.18 and 0.16, respectively).

The brainstem was delineated by all participants and exhibited the lowest IOV (Mean: 25.5 cm^3^, Range: 20.3–29.3 cm^3^, CoV: 0.12). Despite this relatively high concordance, inconsistencies remained regarding the caudal border.

With respect to the OARs, some ROs omitted to delineate organs such as the pituitary gland, the optic chiasm and the temporomandibular joints (TMJ) as shown by the counts in Table 3.

Detailed quantitative data for each contoured volume can be found in the Supplementary Material (Table S1).

## 4. Discussion

### 4.1. Primary Tumor and Low-Risk Elective Nodal Region (PTV1)

**Figure 4:**
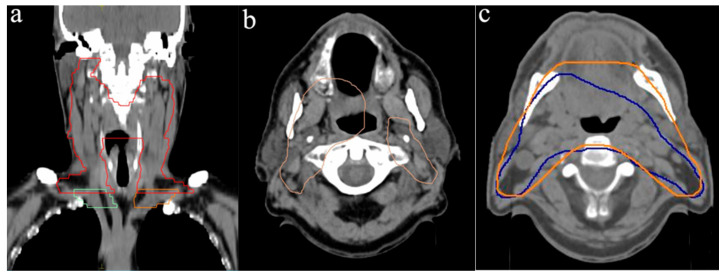
Illustration of the highest discrepancies in PTV delineations: (**a**) Coronal view of a PTV contour (red) not sufficiently including Levels IVb (green and orange) [12], (**b**) PTV outline of one participant with cranial separation, (**c**) Smallest (blue) vs. largest (orange) PTV.

According to the DAHANCA guidelines, cases with N2 lymph node involvement require inclusion of bilateral Levels II and III, as well as ipsilateral Level IV, within the PTV [8]. In alignment with these recommendations, all participants included Levels II and III bilaterally, as well as ipsilateral Level IVa (Table 2). However, the medial supraclavicular Level-IVb left and right was included by only three and four participants, respectively. This discrepancy likely arose because, as noted earlier, the caudal border of the PTV1 was on average located 12 mm above the sternoclavicular joint, leading to an incomplete inclusion of Level IVb (Figure 4a). According to Grégoire et al. the caudal boundary of Level IVb is defined by the cranial edge of the sternal manubrium [12].

In addition, the DAHANCA guidelines recommend inclusion of the submandibular Level Ib when the oral cavity is involved [8]. Despite the absence of such involvement in both panendoscopy and diagnostic imaging, eight participants still included Level Ib bilaterally. Similarly, retropharyngeal lymph nodes (Level VIIa) should be included in cases of posterior pharyngeal wall involvement [8]. Although this was not applicable to the presented case, nearly all radiation oncologists included Level VIIa bilaterally within the PTV1. Notably, only one participant excluded Level VIIa by defining the PTV1 as two separate structures—one encompassing the primary tumor and ipsilateral lymph nodes, and the other covering contralateral lymph nodes (Figure 4b).

Discrepancies were also observed regarding the posterior cervical triangle (Levels Va–Vc). Despite not being recommended as part of the elective lymphatic drainage, between four and seven participants included this region in the PTV. This finding diverges from DAHANCA recommendations but aligns with a study by Chao et al. [14], which analyzed recurrence patterns in 126 patients with head and neck cancer and suggested that T3 and lymph node-positive oropharyngeal carcinoma patients may benefit from elective radiation of contralateral Levels I–V. However, a more recent study by Pouymayou et al. [15] found that progression in these regions was uncommon. Further evidence is provided by Bauwens et al. [16], who compared the distribution of cervical lymph node metastases in HPV-positive and HPV-negative oropharyngeal carcinomas. In HPV-negative, clinically node-positive cases, metastatic involvement was predominantly observed in contralateral Levels II, III, and IV, thereby further supporting the more conservative nodal coverage advocated by the DAHANCA guidelines.

A comparison between the smallest (687 cm^3^) and largest (1098 cm^3^) delineated PTVs still revealed modest agreement at the level of the primary tumor and the infiltrated lymph nodes (Figure 4c). The significant range of PTV, however, resulted from an extensive IOV in inclusion of the medial supraclavicular region. In case of the largest volume, it was extended significantly beyond the lateral border of Level IVb. Anatomically, the lateral boundary of caudal Level IVb is defined by the most lateral aspect of the clavicle [12], yet some participants delineated the volume beyond this reference point, contributing to the observed variation. Finally, the primary contributor to the volumetric range was the delineation of the caudal supraclavicular region. Notable variations were observed in the application of margins, particularly around the infiltration of the base of the tongue and the ipsilateral lymph node metastases.

### 4.2. Primary Tumor and High-Risk Volume (PTV2)

**Figure 5:**
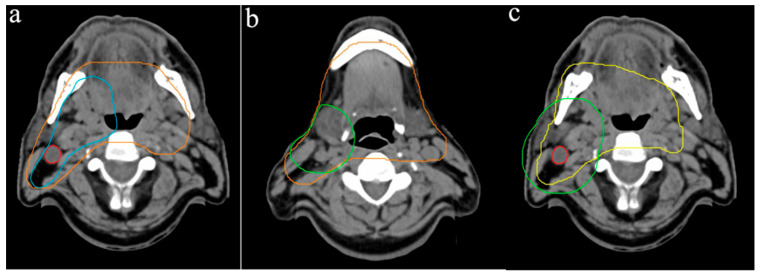
Illustration of PTV2 outlines variations: (**a**) Smallest (light blue) vs. largest (orange) volumes in relation to ipsilateral lymph node metastasis (red), (**b**) Largest volume (orange) in relation to inclusion of submental and submandibular nodes, green contour signifies GTV + a margin of 2 cm, (**c**) Extension of PTV2 (yellow) laterally over the mandibular bone, green contour signifies GTV (red) + a margin of 2 cm.

The volume for primary tumor and high-risk region ranged from 87 cm^3^ to 659 cm^3^, demonstrating a significantly higher CoV compared to the PTV1 (0.55 vs. 0.16), as expected. The smallest volume was generated by defining the high-risk region with minimal margins around the gross tumor volume (GTV), particularly in relation to the ipsilateral lymph node metastases (Figure 5a). In contrast, the largest volume encompassed extensive portions of the oral cavity, along with the contralateral retropharyngeal, upper, and lower jugular regions.

Several participants also included submental and contralateral submandibular lymph nodes within the high-risk volume, despite the absence of pathological findings in either the diagnostic CT or PET scan (Figure 5b). Another notable contributor to largest volume variation was the lateral extension of the contour beyond the mandibular bone to an extent that could not be justified solely by considerations of positioning errors (Figure 5c).

According to standard protocols, the GTV and adjacent high-risk region—where subclinical tumor infiltration is most likely—receive an additional radiation dose. This high-risk region, also referred to as CTV2, is typically defined by a 10 mm isotropic margin around the GTV and may be expanded further for ill-defined tumors [8]. Another consensus guideline by Gregoire et al. propose at least a 5 mm isotropic margin around the GTV for the high-risk volume in patients with T3 oropharyngeal carcinoma [17]. For lymph node metastases smaller than 3 cm, Apisarnthanarax et al. [18] found that tumor infiltration into surrounding tissue remained within 10 mm, supporting this margin for clinical target volume delineation. Figure 3a highlights the ongoing risk of underestimating subclinical tumor spread in clinical practice, particularly since additional safety margins must be applied during PTV delineation to account for setup uncertainties. There was no instruction to apply a specific set-up margin, which also accounted for additional IOV.

However, it is important to note that participants were asked to delineate both the PTV1 and PTV2 without specific guidance on dose prescription. This lack of standardization in dose definition may have exacerbated interobserver variability.

Nevertheless, the substantial interobserver variability observed for target volume delineation in our study is consistent with findings from other contouring analyses, such as the work by Hong et al. [3]. In a head and neck cancer case involving stage III squamous cell carcinoma of the tonsil (T2N1M0) with a pre-defined GTV, the authors reported a similarly high degree of variability for the high-risk CTV, with a coefficient of variation of 0.52. Comparable to our results, the most pronounced discrepancies in nodal coverage were observed in Levels I and V as well as in the contralateral neck. Direct comparison, however, is limited by differences in study design, including the use of a pre-contoured GTV and the differing tumor stage.

### 4.3. Organs at Risk

**Figure 6:**
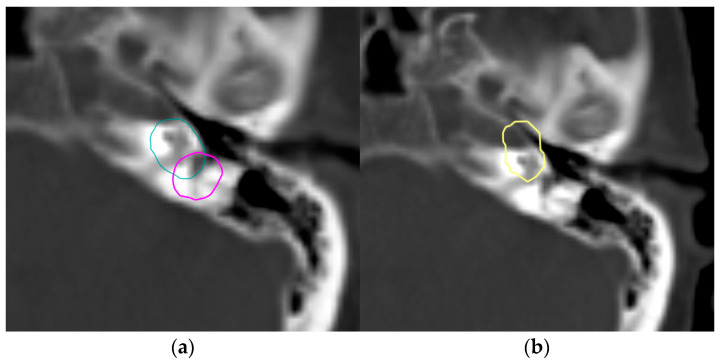
Variation in inner ear outlines: (**a**) Delineation of the semicircular canals rather than the cochlea (pink outline), (**b**) Contour outside the petrous part of the temporal bone. Different colored lines represent the OAR outlines of the respective participants.

Due to a broader interpretation of the inner ear as an OAR, significant variation in contouring was observed (Figure 6a,b). Typically, the cochlea is considered one of the critical OARs in this localization, defined as the bony cavity located anterior to the labyrinth and lateral to the internal auditory canal [19]. The inclusion of the entire petrous portion of the temporal bone, rather than restricting delineation to the cochlea, appears to be a frequent contributor to interobserver variability [20]. Optimal identification of the cochlea is best achieved using CT bone window settings, which may help prevent common sources of error. These include inadvertent contouring of adjacent structures such as the semicircular canals instead of the cochlea (Figure 6a), or excessive ventral extension of the contour over the petrous temporal bone into neighboring structures such as the carotid artery (Figure 6b).

For the optic chiasm, which is typically located ~1 cm superior to the pituitary gland, some participants extended their contours inferiorly into the sella turcica, thereby partially including the pituitary gland.

Concerning the TMJ the variation in axial margins around the OAR and inclusion of the superior portion of the joint within the temporal bone, was possibly generated due to an underutilization of CT bone window settings.

Contours up to 10 mm wide for the optical nerves led to overestimation of the OAR, as the normal optic nerve diameter is only 2–5 mm [21]. Overestimation also occurred when participants inadvertently included structures passing through the superior orbital fissure, such as ophthalmic veins, while attempting to follow the optic nerves into the middle cranial fossa.

For parotid glands some ROs did not consistently delineate the anterior boundary up to the masseter muscle, while others extended the contour beyond the styloid process, failing to recognize it as the medial anatomical boundary [10]. As Nelms et al. have demonstrated, over contouring a parallel organ can result in a significant underestimation of the mean dose, which may increase the risk of more severe xerostomia [2].

According to Brouwer et al. [10], the inferior boundary of the brainstem should be set at the tip of C2; however, only one participant adhered to this guideline, highlighting a need for greater standardization in brainstem delineation. Notably, discrepancies in defining the caudal border of the brainstem were directly linked to variations in the cranial border of the spinal canal. Given the stricter dose constraints for the spinal canal, such inconsistencies could lead to an underestimation of the actual dose received by the spinal cord, potentially impacting treatment safety [19].

Given the small size of the pituitary gland, the 4 mm CT slice thickness likely contributed substantially to the observed interobserver variability. In addition, only seven participants delineated this structure, which may have introduced a selection bias and limited the robustness of the analysis. The incorporation of MRI as well as a high-resolution 3 mm slice CT imaging could have improved visualization, thereby enhancing delineation accuracy and reducing variability. These limitations are not restricted to the pituitary gland but also apply, to varying degrees, to the delineation of other OARs as well as PTV1 and PTV2. Furthermore, the case-study nature of this analysis, based on a single patient dataset, limits the generalizability of the findings to other anatomical presentations and disease extents.

The interobserver variability observed for OAR delineation in the present study is comparable to that reported in previous contouring studies. Nelms et al. [2], for example, reported similar coefficients of variation for the spinal canal and parotid glands, whereas the brainstem exhibited substantially higher variability than observed in our cohort (CoV 0.414 vs. 0.12, respectively). As their analysis was limited to volumetric metrics without an anatomical evaluation, it remains unclear whether discrepancies in defining anatomical boundaries—particularly the caudal border of the brainstem—contributed to the reported variability.

In conclusion, it can be stated that volume delineation remains a complex and time-consuming component of the radiotherapy planning process, influenced by multiple factors such as differences in imaging interpretation, variability in clinical experience and training. Current contouring guidelines, such as those provided by DAHANCA, serve as essential tools for standardizing target volume and OAR delineation. However, their practical application can be limited by variations in anatomical interpretation and the absence of detailed visual examples for complex cases. The usefulness of these guidelines could be further enhanced by supplementing them with anatomical landmark-based standardized contouring templates for target volumes and precise delineation criteria for OARs. Such resources would facilitate clearer understanding of anatomical borders, reduce ambiguity in challenging regions, and promote greater consistency across institutions.

Lastly, recent advances in artificial intelligence have led to the increasing clinical adoption of deep learning-based auto-contouring tools in radiotherapy planning. Multicentre studies, such as that by Pang et al. [22], have demonstrated that AI-assisted contouring can significantly reduce contouring time while also decreasing interobserver variability when combined with clinician review. Although manual editing remains necessary, AI-based auto-segmentation holds substantial potential to improve efficiency and contouring consistency in head and neck radiotherapy.

## 5. Conclusions

This study highlights the persistent interobserver variability in delineating planning target volumes and organs at risk for a patient with oropharyngeal carcinoma, despite the existence of contouring guidelines. This imbalance in contouring practices may affect treatment outcomes by either increasing unnecessary radiation exposure—heightening the risk of toxicities—or failing to adequately cover high-risk regions, potentially compromising tumor control. While certain anatomical regions demonstrated high concordance, significant discrepancies were observed, particularly in the delineation of elective lymph node levels and the margins around the gross tumor volume in defining the high-risk volume. A notable tendency emerged to overestimate the necessity of including elective lymphatic drainage, especially regarding the posterior triangle group, while simultaneously underestimating subclinical tumor extension into surrounding tissues.

Further studies are needed to determine whether these findings reflect a widespread challenge in radiotherapy planning. Such investigations could contribute to refining contouring guidelines and improving standardization to enhance treatment precision and efficacy.

## Figures and Tables

**Table 1:** PTV-Variability: PTV1 = high + low-risk volume, PTV2 = high-risk volume.

	Volume (cm^3^)				Coefficient of Variance	CI_pairs_
	**N**	**Mean**	**SD**	**Range**		
**PTV1**	10	855	138	687–1098	0.16	0.63
**PTV2**	10	386	213	87–659	0.55	0.42

**Table 2:** Inclusion of Lymph node levels (according to Gregoire et al. [12]) in PTV1.

Level	Number of ROs	Levels Covered by All ROs	Levels Covered by No RO
Ia	4	Ib right	VIa left
Ib left	8	IIa left	VIa right
IVa left	9	IIa right	VIb left
IVb left	3	IIb left	VIb right
IVb right	4	IIb right	VIII left + right
Va left	4	III left	IX left + right
Va right	5	III right	Xa left + right
Vb left	4	IVa right	
Vb right	5	VIIa right	
Vc left	5		
Vc right	7		

**Table 3:** Organ-at-risk variability, arranged by most to least variable based on CoV.

	Volume (cm^3^)				Coefficient of Variance
**OAR**	**N**	**Mean**	**SD**	**Range**	
Pituitary gland	7	0.71	0.59	0.18–1.96	0.82
Inner Ear left	10	1.06	0.76	0.12–2.84	0.72
Inner Ear right	10	0.99	0.68	0.11–2.38	0.68
Optic chiasm	9	1.80	1.20	0.36–3.62	0.67
Eye Lens right	10	0.26	0.14	0.15–0.52	0.53
Eye Lens left	10	0.26	0.13	0.14–0.52	0.50
TMJ right	8	1.97	0.76	1.02–3.16	0.39
Optic nerve left	10	1.60	0.54	1.00–2.26	0.34
Optic nerve right	10	1.70	0.54	1.09–2.96	0.32
TMJ left	8	2.01	0.64	1.22–3.03	0.32
Parotid Gland left	10	20.0	5.96	12.0–29.2	0.30
Parotid Gland right	10	19.0	4.62	12.8–26.5	0.24
Spinal canal	10	33.9	6.80	22.7–45.2	0.20
Submandibular Gland left	10	8.66	1.54	5.54–10.4	0.18
Submandibular Gland right	10	8.63	1.37	6.67–11.8	0.16
Brainstem	10	25.5	3.10	20.3–29.3	0.12

## Data Availability

The original contributions presented in the study are included in the Supplementary Material. Further inquiries can be directed at the corresponding author.

## Supplementary Materials

The following supporting information can be downloaded at: https://www.mdpi.com/article/10.3390/curroncol33010039/s1, Table S1: Supplementary data including all contoured volumes.

## Abbreviations

The following abbreviations are used in this manuscript:

CI	Conformity index
CI_pairs_	Conformity index pairs
CoV	Coefficient of variation
CT	Computed tomography
CTV	Clinical target volume
DSC	Dice similarity coefficient
GTV	Gross tumor volume
IOV	Interobserver variability
OAR	Organ at risk
PTV	Planning target volume
PET	Positron emission tomography
RO	Radiation oncologist
SD	Standard deviation
TMJ	Temporomandibular joint
